# Mr. Abel on Hydrophobia

**Published:** 1814-01

**Authors:** C. Abel

**Affiliations:** Halesworth


### *To the Editor of the Medical and Physical Journal.*

SIR,

IN a former Number of your valuable repository, I ventured to call the attention of Dr. Kinglake to Mr. Borrett's paper on Hydrophobia, and to claim for its author the merit of priority in remarking with distinctness the analogy between that disease and Gastritis; and my best thanks are due to Dr. Kinglake, for the very polite and candid manner in which he met my appeal. I have also had the good fortune to elicit from Dr. Adams several interesting remarks on Mr. Borrett's communication, to some of which I shall in this paper attempt to reply.

In sending for your insertion my observations on Dr. K.'s claim to originality of opinion on Hydrophobia, I was not ignorant that several authors, in common with Dr. Adams, had observed the generally inflammatory form of some of its symptoms, especially Boerhaave, Van Swieten, and Morgagni, and from whose works it may not be uninteresting to some of your readers, if I adduce a few passages in illustration of their views of this formidable malady. The following passage forms a part of Boerhaave's eleven hundred and fortieth axiom: "Dissectio cadaverum docerit plerumque organa deglutiendi utrumque inflammata,"* &c. Again, in Aphorism 1142: "Dein hinc vitium nasci sanguini, humoribusque, quod inflammationi gangrenosæ quasi proximum sit; sedem vera mali primum circa stomachum, et vicina hærere." As to the methodus medendi, this author is particularly striking. In Aphor. 1144, the following remark occurs: "Apparet maxime probabile, et paucis experimentis confirmatum, sequentia fieri debere: 1. Statim post prima signa invadentis mali morbus tractandus *ut summus inflammatorius*, mittendo sanguinem *ex lato vulnere magni vasis ad animi deliquium usque*," &c. Van Swieten, in his commentaries on these passages, affords sufficient proof that he had closely noticed the evidences offered by different histories, of the inflammatory nature of rabies canini; of which the following examples may be given:—"Alibi legitur inflammationi ventriculi accessisse hydrophobiam cum convulsionibus, a quibus malis tamen æger ille audacter repetitis sanguinis emitionibus evasit."—The author here alludes to a case detailed in the first volume of Medical-Essays. And in another passage this writer is equally explicit:—"Tota autem morbi historia, et aliqui successus in hoc

* Van Sw. Oper. An. p. 561. Ibid. p. 566.

morbo morbo curando, videntur suadere, morbum hunc tractare debere ut inflammationem summam, admodum periculosam, et subito in gangrænam tendentem."*

That Morgagni was aware of similar facts, the greater part of his eighth letter affords clear testimony; in particular, he cites three cases in which symptoms of hydrophobia supervened after drinking cold water.

But although remembering the passages just quoted, I knew of no author who like Mr. B. had with confidence recommended the means which have recently proved so successful; a confidence founded not merely on partial or complete success in the treatment of analogous disorders, but the result of a minute investigation of the symptoms and dissection of a case, under his personal observation; a confidence infused into the minds of several of his readers, and which had prepared them, should a case fall under their care, to treat it "ut inflammationem summam."

With respect to the appearances of the stomach on dissection, as observed by Mr. B., I am disposed to consider them as much more attributable to inflammation than to digestion, and for the following reasons. Mr. Hunter, in his description of a digested stomach, observes,† "The sound portions (of the stomach) will appear soft, spongy, and granulated, and without distinct blood-vessels, opake, and thick; while the others will appear smooth, thin, and more transparent; and the vessels will be seen ramifying in its substance, and upon *squeezing* the blood which they contain from the larger branches to the smaller, it will be found to pass out at the digested ends of the vessels, and to appear like drops on the inner surface."

From the above quotation it is evident, that no appearance of red points is visible on a simple inspection of a digested stomach; but in that described by Mr. Borrett, such points studded a part of its inner surface, and consequently, even supposing an erosion of the extremities of the vessels could only arise from a turgescent state of them, the effect of inflammation; for, under common circumstances, the blood after death is accumulated in their larger branches, and must, as expressed by Mr. Hunter, be *squeezed* thence into their minuter ramifications, to exhibit the appearance in question. Neither can I find, in Mr. Hunter's or Dr. Baillie's account of a digested stomach, any mention of an appearance similar to the dark-colored patches noticed in Mr. B.'s

* Comment. p. 537. De Caus. et Sed. Morb. Epist. iii. Art. 32.

† An. Economy, p. 229.

history, history; but which are so frequently not within descriptions of stomachs unquestionably inflamed, and which appear to depend on an extravasation of blood under the mucous membrane; in proof of which I shall quote a part of Mr. Brodie's excellent statement of the changes produced by arsenic on the inner surface of the stomach, and which agrees remarkably with the effect attributed by Mr. Borrett to the hydrophobic poison. *"The inflamed parts are in general universally red, at other times they are red only in spots. The principal vessels leading to the stomach and intestines are tinged with blood; but the inflammation is usually confined to the mucous membrane of these viscera, which assumes a florid red color, becomes soft and pulpy, and is separable without much difficulty from the cellular coat, which has its natural appearance. In some instances there are small spots of extravasated blood on the inner surface of the mucous membrane, or between it and the cellular coat, and this occurs independently of vomiting."

The destruction of the cuticle of the lower part of the œsophagus, reaching to the orifice of the cardia, beyond which it does not appear to have extended, cannot, I conceive, be fairly attributed to the action of the gastric fluid; for it is equally difficult to comprehend how this should escape into the gullet, and if there, why it should act more powerfully on that part than on the stomach itself. The gastric juice could only penetrate to the œsophagus through the valvular structure of the cardiac orifice of the stomach, which, in consequence of its longer exposure to this solvent, ought to have suffered more than the œsophagus. Neither Mr. Hunter, nor Dr. Baillie, mentions the liability to destruction of the inner membrane of the œsophagus from such a cause; and it seems highly improbable that so important a circumstance should have been overlooked by those accurate observers. I am, Sir,

*Halesworth,* Your most obedient Servant,

*Nov.* 13, 1813. C. ABEL.

---

*To the Editor of the Medical and Physical Journal.*

SIR,

THE following case occurred to a patient under my care in the work-house of this parish, the particulars of which I beg leave to relate.

Mrs. Thomas, aged 47 years, applied to me, a few days after

---

* Phil. Trans. for 1812, p. 205; or Nichols. Journ. vol. xxxiii. p. 265.

